# Association of Sweet's Syndrome and Systemic Lupus Erythematosus

**DOI:** 10.1155/2011/242681

**Published:** 2011-11-14

**Authors:** J. L. Barton, L. Pincus, J. Yazdany, N. Richman, T. H. McCalmont, L. Gensler, M. Dall'Era, K. H. Fye

**Affiliations:** Division of Rheumatology, Department of Medicine, University of California, San Francisco, CA 94143, USA

## Abstract

Sweet's syndrome is an acute febrile neutrophilic dermatosis which usually presents as an idiopathic disorder but can also be drug induced, associated with hematopoetic malignancies and myelodysplastic disorders, and more, infrequently, observed in autoimmune disorders. Sweet's syndrome has been reported in three cases of neonatal lupus, three cases of hydralazine-induced lupus in adults, and in nine pediatric and adult systemic lupus erythematosus (SLE) patients. We describe three additional adult cases of Sweet's associated with SLE and provide a focused review on nondrug-induced, nonneonatal SLE and Sweet's. In two of three new cases, as in the majority of prior cases, the skin rash of Sweet's paralleled underlying SLE disease activity. The pathogenesis of Sweet's remains elusive, but evidence suggests that cytokine dysregulation may be central to the clinical and pathological changes in this condition, as well as in SLE. Further research is needed to define the exact relationship between the two conditions.

## 1. Introduction

Sweet's syndrome represents an acute neutrophilic dermatitis, often with associated fever [1], first described by Sweet in 1964 [2]. Although the syndrome often presents in idiopathic fashion, it can also be induced by medications and has been associated with hematopoietic malignancies and myelodysplastic disorders. It has also been observed in association with certain autoimmune disorders, such as Sjogren's syndrome. Nine patients with both Sweet's syndrome and systemic lupus erythematosus (SLE) have been previously reported [3–10]. Because the diagnosis of Sweet's syndrome can be challenging, particularly when associated with other connective tissue disorders such as SLE, a set of diagnostic criteria were proposed initially by Su and Liu [11] and then revised by Von den Driesch [12]. The diagnosis is based upon the presence of two major and two of the four minor criteria. The two major criteria are (1) abrupt onset of painful erythematous plaques or nodules and (2) histopathologic evidence of a dense neutrophilic infiltrate. The minor criteria include (1) fever >38 degrees centigrade; (2) presence of a malignancy or connective tissue disease; (3) dramatic response to corticosteroids or potassium iodide therapy; (4) an elevated erythrocyte sedimentation rate or leukocytosis.

 We herein report our experience with Sweet's syndrome among adult patients in the Lupus Clinic at the University of California, San Francisco, along with a review of the literature.

## 2. Case 1

A 26-year-old previously healthy Japanese female presented with nine days of myalgias, subjective fevers, and soaking night sweats. Four days prior, she developed multiple erythematous nodules on her face, chest, abdomen, and upper and lower extremities bilaterally. She complained of sore throat, nonproductive cough, painless oral ulcers, hand swelling, Raynaud's phenomenon, erythroderma, and abdominal bloating. She denied illicit or prescribed drug use, drug allergies, alcohol, or tobacco. She emigrated from Japan at age eight and received a BCG vaccination as a child. She reported recent travel to Europe and was sexually active with one partner. 

**Figure 1:**
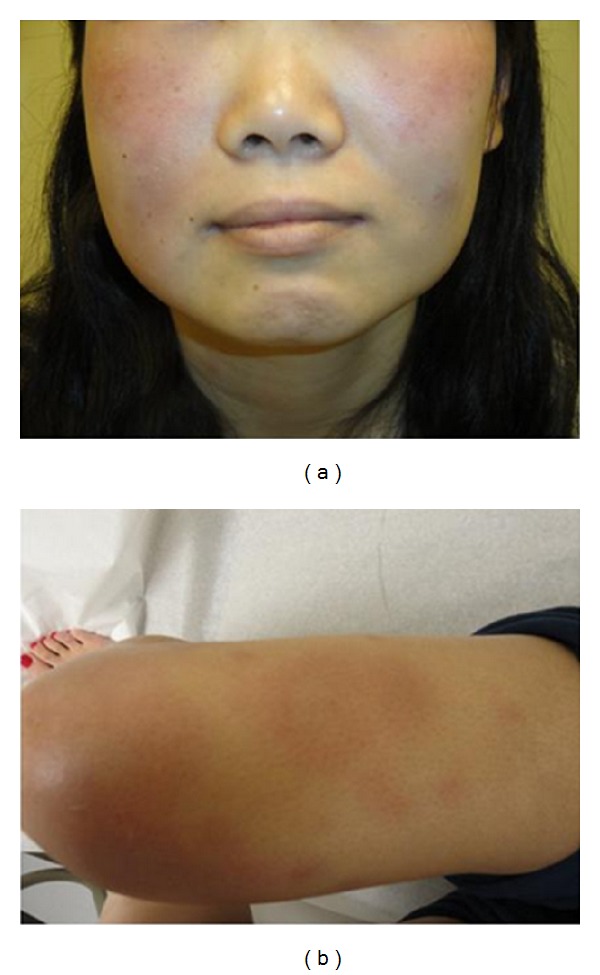
Multiple 2-3 cm tender, erythematous, subcutaneous nodules were present over (a) the face and (b) lower extremities.

On physical examination, the patient was normotensive and afebrile. There was a tender right submandibular node and diffuse swelling of the hands without synovitis. Multiple 2-3 cm tender, erythematous, subcutaneous nodules were present over the face, chest, abdomen, and upper and lower extremities bilaterally (Figures 1(a) and 1(b)). She subsequently developed a fever of 39.5 Celsius. Initial laboratory tests included a hematocrit of 30.9%, an LDH of 329 iu/L, a positive Coombs antibody assay, an erythrocyte sedimentation rate of 58 mm/hr Westergren, a c-reactive protein of 182 mg/L (normal <6.3 mg/L), and a prolonged partial thromboplastin time of 38.7 sec (normal 20.9–33.6). Histochemical staining of the skin biopsy did not reveal organisms. A CT scan of the chest revealed interlobular septal and bronchial wall thickening of multiple lobes and patchy ground glass opacities suggestive of pulmonary edema or atypical or viral pneumonia. Further laboratory testing revealed an antinuclear antibody titer >640, speckled with positive anti-Smith and anti-Sm/RNP antibody assays. Complement levels and urinalysis were normal. A tuberculin skin test was positive to 14 mm at 48 hours. A quantiferon gold assay was indeterminate. A skin biopsy from her left thigh showed a relatively sparse interstitial inflammatory infiltrate composed of neutrophils, lymphocytes, and histiocytes. In addition, there were some foci of leukocytoclastic vasculitis. Neutrophils were also found in the subcutaneous tissue.

The patient was treated with prednisone 40 mg and hydroxychloroquine 400 mg daily with a dramatic response. Dapsone was later added in an effort to taper the prednisone. She was also treated for latent tuberculosis with four months of rifampin.

## 3. Case 2

A 23-year-old Asian-American female with a history of SLE presented to the emergency department with a five-day history of diffuse myalgias, fatigue, dizziness, and three days of erythematous papules and plaques which began on the left side of the face and then spread to the upper trunk, arms and legs. On presentation, temperature was 38.9 Celsius. Routine blood tests, chest radiograph, rapid strep, blood cultures, and throat swabs for gonorrhea and Chlamydia were negative. An erythrocyte sedimentation rate was 54 mm/hr Westergren. The patient was instructed to follow up in the lupus clinic. Manifestations of SLE diagnosed at age 20 included malar rash, arthritis, pericarditis, Raynaud's, Coombs positive autoimmune hemolytic anemia, antinuclear antibody titer >640 speckled, and elevated titers of anti-dsDNA antibodies. She was a nursing student, denied tobacco, alcohol, or illicit drug use, and was sexually active with one partner. Medications included hydroxychloroquine 400 mg, prednisone 3 mg, and calcium with vitamin D.

The following day in clinic, the patient complained of painful erythematous papules and plaques as well as intermittent discomfort over the left chest which worsened with deep inspiration, palpitations, and Raynaud's phenomenon. She denied hair loss, arthralgias, dyspnea, oral ulcerations, or new medications. Physical examination revealed an afebrile, normotensive female with tachycardia (pulse 114), cobblestone appearance and erythema of bilateral tonsils with slight exudate, and shotty cervical lymphadenopathy. Cardiac and chest exam were normal. Skin exam revealed large erythematous, tender, and indurated papules and plaques over the face with smaller lesions scattered over the upper chest, back, upper legs, and arms.

Repeat laboratories and skin biopsy were performed. Hemoglobin dropped from 13.7 g/dL to 10.9 g/dL over two days. A skin biopsy of the right upper arm revealed an extensive interstitial infiltrate of neutrophils and histiocytes accompanied by leukocytoclastic debris, suggestive of Sweet's syndrome. A skin biopsy over the chin 17 months later showed neutrophilic dermatitis with leukocytoclasis. Treatment with higher dose prednisone and azathioprine resulted in a good response. However, as indicated by the repeat biopsy, the rash recurred as prednisone was tapered.

## 4. Case 3

**Figure 2:**
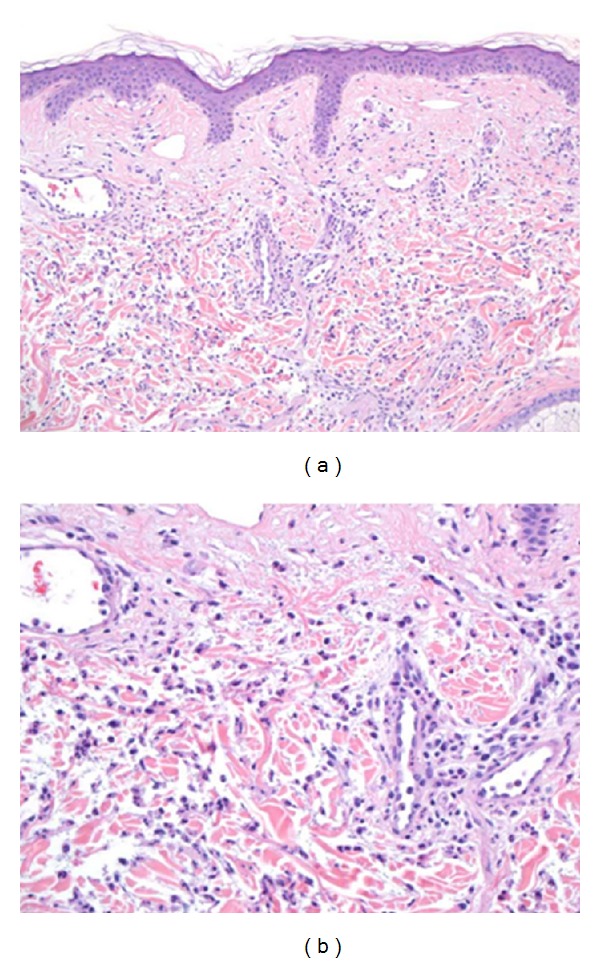
(a) Low magnification showing a diffuse dermal infiltrate of neutrophils accompanied by leukocytoclastic debris. (b) High magnification highlighting the neutrophils.

A 23-year-old Asian male with a history of SLE diagnosed at age 16 presented for followup with his nephrologist. He complained of persistent right facial swelling for 12 months. The swelling was limited to the skin over the right parotid gland and was associated with intermittent pruritic vesicles. Manifestations of SLE included a discoid rash and lupus nephritis. Abnormal serologies included antinuclear antibody titer >640 diffuse and elevated titers of anti-dsDNA antibodies. Medications included prednisone 5 mg, enalapril 5 mg, and tacrolimus 2 mg twice daily. The patient was an engineer, used tobacco and alcohol occasionally, and was not sexually active. On examination, the patient was normotensive and afebrile. He had slight warmth and erythema over the right parotid gland without tenderness. Laboratory data revealed an elevated anti-dsDNA antibody titer of 271 iu/mL (250 iu/mL one month prior). Complete blood count, complement levels, creatinine, urinalysis, and c-reactive protein were all normal. A fine needle aspiration of the parotid gland revealed a mixed population of lymphocytes with scattered lymphoepithelial islands, diagnostic of lymphoepithelial sialadenitis. Skin biopsy of the right cheek showed a diffuse infiltrate of neutrophils and leukocytoclastic debris consistent with Sweet's syndrome (Figures 2(a) and 2(b)). The patient was started on hydroxychloroquine 400 mg and prednisone with an excellent response.

## 5. Discussion

While Sweet's syndrome can occur in idiopathic fashion, it can also be drug induced, associated with malignancy [13], or connective tissue diseases. Concurrent Sweet's disease and SLE are exceedingly rare. Only two other papers reported Sweet's syndrome as an initial presentation in adult SLE [6, 8]. Our Case 1 is the third. Three cases of Sweet's syndrome-like nonbullous neutrophilic dermatosis and neonatal lupus have been reported [14, 15], as well as three cases of hydralazine-induced SLE and Sweet's [16–18], but will not be discussed here. In addition, cases with some features of SLE that did not meet full criteria for SLE were excluded from this paper.

The skin lesions in Sweet's syndrome typically start as erythematosus papules, plaques, and nodules. The lesions can take on a pseudovesicular or pseudopustular appearance, and sometimes fully formed vesicles or pustules develop. The lesions are usually painful, and typical sites include the head, neck, and upper extremities, although lesions can be found anywhere. Lesions usually develop abruptly, resolve over 1–3 months, and can recur in 30% of patients [19]. One of the two major criteria as outlined by Von den Driesch is a typical cutaneous rash [12]. Indeed, all three patients described herein had a rash compatible with Sweet's syndrome. Both Cases 1 and 2 had lesions on the face, upper trunk, arms, and legs. Patient 3 had erythematosus swelling on his right face with vesicles within it for 12 months. Although the time course of this patient's lesion was atypical for Sweet's, the vesicles waxed and waned, suggesting resolution and recurrence, as can be seen. 

The prototypical histopathologic findings in Sweet's syndrome include a diffuse dermal infiltrate of neutrophils accompanied by leukocytoclasis with overlying papillary dermal edema and, in some cases, extending into the subcutaneous septa. Although older studies reported that leukocytoclastic vasculitis is not seen [20], more recent reports have convincingly demonstrated that leukocytoclastic vasculitis can occasionally be found [21]. The skin biopsies from all patients described herein were compatible with Sweet's syndrome as they exhibited a dermal neutrophilic infiltrate accompanied by leukocytoclasis (Table 1). In Cases 2 and 3, the neutrophilic infiltrate was diffuse and extensive, although in Case 1 it was relatively sparse. Additional histopathologic findings in Case 1 included extension of the neutrophilic infiltrate into subcutaneous septa and accompanying leukocytoclastic vasculitis. 

As posited by Gleason and colleagues [9], infiltrates that are less dense than what is typically observed in Sweet's disease may be classified as “neutrophilic dermatosis of LE (lupus erythematosus).” In fact, our Case 1 probably best fits into this classification. It seems most plausible that Sweet's syndrome and neutrophilic dermatosis of LE are related and on a spectrum. It is important for rheumatologists and dermatologists to recognize the possibility, as suggested by Gleason, that what appears to be a distinct entity (Sweet's syndrome) may in fact be a variant of cutaneous LE and may represent the first manifestation of the systemic disease, as in Case 1. 

These new cases, combined with nine others in the literature, raise several important questions. Does Sweet's syndrome occur as an independent process, or is it merely a manifestation of the underlying autoimmune disease and an underrecognized variant of cutaneous LE? In two out of three new cases, the rash consistent with Sweet's syndrome paralleled underlying SLE activity. In Case 1, the patient had concomitant oral ulcerations, hand swelling, and a Coombs positive hemolytic anemia. In Case 2, the patient had serositis and a Coombs positive hemolytic anemia. The nine other cases in the literature reflect a similar pattern of Sweet's presenting along with manifestations of active SLE. 

Although the pathogenesis of Sweet's syndrome remains elusive, evidence suggests that cytokine dysregulation may be central to the clinical and pathological changes. Studies have implicated a variety of cytokines, including IL-1, IL-3, IL-6, and IL-8 and G-CSF, GM-CSF and interferon gamma [22, 23]. Some of these cytokines, such as IL-6, may also play a role in SLE through a variety of mechanisms, including B cell stimulation and induction of acute phase reactants [24]. Similarly, interferon gamma may be involved in the pathogenesis of SLE. In one series of experiments, NZB mice treated with interferon developed hemolytic anemia earlier and had a significant increase in renal immune complex deposition and renal failure [25]. This preliminary evidence suggests that cytokine dysregulation plays a role in both conditions. It may be that in certain patients during a SLE flare, some cytokines implicated in eliciting Sweet's syndrome are released, resulting in clinical manifestations on the spectrum of Sweet's syndrome and Sweet's syndrome-like neutrophilic dermatitis. 

Like other cases in the literature, our patients responded dramatically to corticosteroids, which are the mainstay of therapy for Sweet's syndrome. Other immunosuppressive treatments, including cyclosporine, have also been used successfully. Additional treatments include potassium iodide, dapsone, colchicine, indomethacin, and clofazimine.

In summary, Sweet's syndrome is a neutrophilic dermatoses that may be more commonly associated with SLE than previously suspected because of the role of cytokine dysregulation in the pathogenesis of both conditions. Sweet's disease observed in the setting of lupus may also be classified as “neutrophilic dermatosis of LE” and may be the first manifestation of SLE. Further research is needed to define the exact relationship between the two conditions and to enhance awareness among both rheumatologists and dermatologists.

## Figures and Tables

**Table 1:** Demographic and clinical characteristics of SLE patients at time of presentation with Sweet's.

Patient	Gender	Age	Race/ethnicity	Clinical manifestations of SLE at time of Sweet's presentation	Serologies	Histopathology	Treatment	Response
1	Female	26	Asian	Oral ulcers, Raynaud's, hemolytic anemia	ANA, anti-Sm, anti-Sm/RNP	Sparse interstitial neutrophilic dermatitis	Prednisone 40 mg, hydroxychloroquine	Responsive to treatment
2	Female	23	Asian	Malar rash, pericarditis, arthritis, hemolytic anemia	ANA, anti-dsDNA, anti-La,	Neutrophilic dermatitis	Prednisone	Responsive to treatment
3	Male	23	Asian		ANA, dsDNA	Neutrophilic dermatitis	Hydroxychloroquine, prednisone	Responsive to treatment
Goette 1985 [7]	Male	67	NA	Arthritis, malar, and discoid rashes, leukopenia, thrombocytopenia	ANA	Prominent dermal edema, extensive infiltration with intact and karyorrhectic PMNs	Prednisone 30–40 mg, quinacrine hydrochloride 100 mg	Responsive to treatment
Choi and Chung, 1999 [5]	Female	13	Asian	Malar rash, oral ulcers, leukopenia, thrombocytopenia, nephritis	ANA, anti-Sm, anti-dsDNA	Neutrophilic dermatosis	Pulse methylprednisolone	Responsive to treatment
Burnham and Cron, 2005 [10]	Female	14	African American	Arthritis, oral ulcer, leukopenia	ANA, anti-dsDNA, anti-Ro, anti-La, anti-SM/RNP	Marked edema of papillary dermis with early subepidermal bullae, PMNs and karyorrhexis	NA	NA
Hou et al., 2005 [8]	Female	38	Asian	Arthritis, proteinuria >0.5 mg/day,	ANA, anti-Ro, anti-La	Neutrophilic dermatosis	Prednisolone 20 mg	Responsive to treatment
Gleason et al., 2006 [9]	Female	16	NA	Arthritis, serositis, leukopenia	ANA, anti-dsDNA	Neutrophilic dermatosis	Hydroxychloroquine, prednisone	Responsive to treatment
Gleason et al., 2006 [9]	Female	35	NA	Arthritis, hematuria (lupus nephritis on prior biopsy)	ANA, anti-dsDNA, anti-Ro	Neutrophilic dermatosis	Corticosteroids, dapsone, azathioprine	Responsive to treatment
Camarillo et al., 2008 [3]	Female	9	NA	Lymphopenia, (mesangial glomerulonephritis prior)	ANA, anti-dsDNA, anti-RNP	Interstitial dermal infiltrate of histiocytelike cells, lymphocytes and segmented neutrophils and leukocytoclastic debris	Methylprednisolone, dapsone	Responsive to treatment
Fernandes et al., 2009 [6]	Male	25	NA	Leukopenia, proteinuria, malar rash	ANA, anti-Sm/RNP, anti-dsDNA	Neutrophilic dermatitis	Prednisone	Responsive to treatment
Gollol-Raju et al., 2009 [4]	Female	23	NA	Malar rash (lupus nephritis previously)	ANA	Acute inflammatory cells with overlying keratin debris, abundant neutrophilic dust	Corticosteroids	Responsive to treatment

## References

[1] Storer JS, Nesbitt LT, Galen WK, DeLeo VA (1983). Sweet’s syndrome. *International Journal of Dermatology*.

[2] Sweet RD (1964). An acute febrile neutrophilic dermatosis. *British Journal of Dermatology*.

[3] Camarillo D, McCalmont TH, Frieden IJ, Gilliam AE (2008). Two pediatric cases of nonbullous histiocytoid neutrophilic dermatitis presenting as a cutaneous manifestation of lupus erythematosus. *Archives of Dermatology*.

[4] Gollol-Raju N, Bravin M, Crittenden D (2009). Sweet’s syndrome and systemic lupus erythematosus. *Lupus*.

[5] Choi JW, Chung KY (1999). Sweet’s syndrome with systemic lupus erythematosus and herpes zoster. *British Journal of Dermatology*.

[6] Fernandes NF, Castelo-Soccio L, Kim EJ, Werth VP (2009). Sweet syndrome associated with new-onset systemic lupus erythematosus in a 25-year-old man. *Archives of Dermatology*.

[7] Goette DK (1985). Sweet’s syndrome in subacute cutaneous lupus erythematosus. *Archives of Dermatology*.

[8] Hou TY, Chang DM, Gao HW, Chen CH, Chen HC, Lai JH (2005). Sweet’s syndrome as an initial presentation in systemic lupus erythematosus: a case report and review of the literature. *Lupus*.

[9] Gleason BC, Zembowicz A, Granter SR (2006). Non-bullous neutrophilic dermatosis: an uncommon dermatologic manifestation in patients with lupus erythematosus. *Journal of Cutaneous Pathology*.

[10] Burnham JM, Cron RQ (2005). Sweet syndrome as an initial presentation in a child with systemic lupus erythematosus. *Lupus*.

[11] Su WPD, Liu HNH (1986). Diagnostic criteria for Sweet’s syndrome. *Cutis*.

[12] Von den Driesch P (1994). Sweet’s syndrome (acute febrile neutrophilic dermatosis). *Journal of the American Academy of Dermatology*.

[13] Kaiser R, Connolly K, Linker C, Maldonado J, Fye K (2008). Stem cell transplant for myelodysplastic syndrome-associated histiocytoid Sweet’s syndrome in a patient with arthritis and myalgias. *Arthritis Care and Research*.

[14] Barr KL, O’Connell F, Wesson S, Vincek V (2009). Nonbullous neutrophilic dermatosis: sweet’s syndrome, neonatal lupus erythematosus, or both?. *Modern Rheumatology*.

[15] Satter EK, High WA (2007). Non-bullous neutrophilic dermatosis within neonatal lupus erythematosus. *Journal of Cutaneous Pathology*.

[16] Ramsey-Goldman R, Franz T, Solano FX, Medsger TA (1990). Hydralazine induced lupus and Sweet’s syndrome report and review of the literature. *Journal of Rheumatology*.

[17] Sequeira W, Polisky RB, Alrenga DP (1986). Neutrophilic dermatosis (Sweet’s syndrome). Association with a hydralazine-induced lupus syndrome. *American Journal of Medicine*.

[18] Servitje O, Ribera M, Juanola X, Rodriguez-Moreno J (1987). Acute neutrophilic dermatosis associated with hydralazine-induced lupus. *Archives of Dermatology*.

[19] Bolognia J, Jorizzo JL, Rapini RP (2008). *Dermatology*.

[20] Goldman GC, Moschella SL (1971). Acute febrile neutrophilic dermatosis (Sweet’s syndrome). *Archives of Dermatology*.

[21] Malone JC, Slone SP, Wills-Frank LA (2002). Vascular inflammation (vasculitis) in Sweet syndrome: a clinicopathologic study of 28 biopsy specimens from 21 patients. *Archives of Dermatology*.

[22] Elinav H, Maly A, Ilan Y, Rubinow A, Naparstek Y, Amital H (2004). The coexistence of Sweet’s syndrome and Still’s disease—is it merely a coincidence?. *Journal of the American Academy of Dermatology*.

[23] Giasuddin ASM, El-Orfi AHAM, Ziu MM, El-Barnawi NY (1998). Sweet’s syndrome: is the pathogenesis mediated by helper T cell type 1 cytokines?. *Journal of the American Academy of Dermatology*.

[24] Youinou P, Jamin C (2009). The weight of interleukin-6 in B cell-related autoimmune disorders. *Journal of Autoimmunity*.

[25] Heremans H, Billiau A, Colombatti A (1978). Interferon treatment of NZB mice: accelerated progression of autoimmune disease. *Infection and Immunity*.

